# Professional Titles, Pleasant and Unpleasant

**Published:** 1915-03-20

**Authors:** 


### The Hospital

The Workers' Newspaper of Administrative Medicine and Institutional Life, Administration, National Insurance and Health.

No. 1500, Vol. LVII. SATURDAY, MARCH 20, 1915. PRICE ONE PENNY.

### PROFESSIONAL TITLES, PLEASANT AND UNPLEASANT.

The technical terms in which medical practitioners describe themselves and one another often prove a stone of stumbling to the general public. Between "Mr." and "Dr.," Member of this and Fellow of that—not to speak of the presentation of these distinctions in alphabetical configuration—there is little wonder that confusion results. The terms, in some instances, can hardly be pronounced, much less interpreted. Of recent years there has been an increasing tendency to overcome the difficulty—or rather to avoid it—by applying to every medical practitioner the title of "Doctor," this term being used as a description of occupation and not as an index of academic rank. This is not altogether welcome to the consulting surgeons, who prefer the designation "Mr." on the ground, presumably, that their claims to attention are those of the expert and not of the learned; nor does it quite suit the taste of some who would fain exalt their youthful academic triumphs to the level of a permanent superiority.

Nevertheless, the term "Doctor," used as just described, has a considerable public convenience, and laymen may well be excused from an active interest in professional shibboleths. There is this further advantage: that the title "Doctor" enjoys, at least implicitly, legal protection, in the sense that, used by a non-registered person engaged in medical or surgical practice for gain, it would lead to prosecution and penalties. Hence in practical affairs the term does mark a distinction between persons who are qualified in the legal sense of this word and those who are unqualified. There is, of course, no law which forbids unqualified persons to practise. What the law demands is that such persons shall not mislead the public by professing or pretending to be qualified. To adopt the title of "Doctor" is a profession of qualification, and, therefore, in the circumstances contemplated, would be a legal offence. It follows, that the use of this word as applied to medical practitioners generally, however incorrect academically, has the advantage of marking a clear distinction between persons who have received a definite technical training and others who achieve greatness by a less tedious method.

A contrast to the position just defined is presented by two other terms largely used by the public and adopted—unhappily, as it seems to us—in certain professional circles. We refer to the words "specialist" and "consultant." Whatever may be their merits, these titles certainly do not enjoy, either explicitly or implicitly, any established legal status or meaning. Far from indicating necessarily the possession of a registrable medical qualification, they are open to any pretender or quacksalver of the day and hour. For the time being they seem to be particularly cultivated by persons who apply their native genius to the cure of obesity, to the restoration of faded or fallen locks, and to the cultivation of the mysteries by which female beauty in all its varied exhibition is promoted or conserved. Some of the individuals concerned with these ambitions call themselves "specialists," others announce themselves as "consultants," while in a third group the members utilise both terms, and thus the "consultant specialist" is born. That all this is strictly within the four corners of the law there can be no doubt. And, at least so far as we are concerned, there is no desire that new legal powers should be invoked. On the contrary, we accept with calm, and even with gratitude, announcements which help to brighten the breakfast-table, and which assure us of the presence in our midst of persons devoted to scientific studies and distinguished by so large a measure of eminence, tact, consideration, and good will. It is, however, certain that members of the medical profession do not compete in these exercises; nor do they make public proclamation of such humble virtues as they chance to possess. Surely, then, it is obvious that they ought to avoid descriptive terms which may possibly lead to confusion. By keeping certain courses of study and passing the prescribed examinations a moderately industrious and capable person may legally and legitimately attain to the position of a "physician" or "surgeon," while genius, knowing nothing of these restrictions, becomes at a bound a "consultant" and a "specialist." Let plain men and women, therefore, keep to the modest titles which the law bestows upon them and leave pretentious claims to more strident trumpeters. In a word, the medical profession has a kingdom of its own, where none other may enter and where it may dwell safely and without challenge. It is surely well, in these circumstances, to leave strange lands to the gods which boast their possession.

It may, perhaps, be urged that the course here suggested would mean difficulty in indicating the status and work of those who confine themselves to particular branches of practice, or who undertake to see patients only in consultation with other practitioners. But it is surely as easy to speak of an ophthalmic surgeon or an aural surgeon as it is of a surgeon-dentist, and can anyone wish a more ambitious title than consulting physician or consulting surgeon? Such terms have both legal value and professional traditions, while the new-fangled phrases, ranging from neurologist to proctologist, can claim neither the one nor the other. In the selection of professional titles it is well to avoid terms which proclaim narrowness of outlook, or which are equally the property of those who are without the pale.

